# Polymer Stabilization of Uniform Lying Helix Texture in a Bimesogen-Doped Cholesteric Liquid Crystal for Frequency-Modulated Electro-Optic Responses

**DOI:** 10.3390/ma15030771

**Published:** 2022-01-20

**Authors:** Chia-Hua Yu, Po-Chang Wu, Wei Lee

**Affiliations:** 1 College of Photonics, National Yang Ming Chiao Tung University, Guiren Dist., Tainan 711010, Taiwan; chyu1029@gmail.com; 2 Institute of Imaging and Biomedical Photonics, College of Photonics, National Yang Ming Chiao Tung University, Guiren Dist., Tainan 711010, Taiwan; jackywu@nycu.edu.tw

**Keywords:** cholesteric liquid crystal, uniform lying helix, polymer network, frequency modulation, electro-optic response, mesogenic dimer, flexoelectric effect, dielectric effect

## Abstract

A polymer network (PN) can sustain the uniform lying helix (ULH) texture in a binary cholesteric liquid crystal (LC) comprising a calamitic LC and a bimesogenic LC dimer. Upon copolymerization of a bifunctional monomer with a trifunctional monomer at a concentration of 5 wt% to create the desired polymer network structure, the PN-ULH was obtained with high stability and recoverability even when cycles of helical unwinding-to-rewinding processes were induced after the electrical or thermal treatment. Utilizing dielectric spectroscopy, the flexoelectric-polarization-dominated dielectric relaxation in the PN-ULH state was characterized to determine two frequency regions, *f* < *f*_flexo_ and *f* > *f*_di_, with pronounced and suppressed flexoelectric effect, respectively. It is demonstrated that the cell in the PN-ULH state can operate in the light-intensity modulation mode by the flexoelectric and dielectric effects at *f* < *f*_flexo_ and phase-shift mode by the dielectric effect at *f* > *f*_di_. Moreover, varying the voltage frequency from *f* < *f*_flexo_ to *f* > *f*_di_ results in a frequency dispersion of transmittance analogous to that of flexoelectric-polarization-dominated dielectric relaxation. The unique combination of the bimesogen-doped cholesteric LC with a stable and recoverable PN-ULH texture is thus promising for developing a frequency-modulated electro-optic device.

## 1. Introduction

Uniform lying helix (ULH) is a kind of cholesteric liquid crystal (CLC) texture whose helical pitch is shorter than the wavelengths of visible light and is uniformly aligned along a preferred direction in the substrate plane of confining geometry. When a voltage is applied across the ULH helix, two types of helix reorientation can be generated by voltage-induced flexoelectric and dielectric effects. At a moderate voltage where the dielectric effect is negligible, the flexoelectric coupling of LC molecules with the electric field produces a periodic splay-bend deformation, giving rise to the in-plane deviation of the ULH optic axis. The magnitude of such a deviation angle induced by the flexoelectric effect is in principle a linear function of the voltage amplitude and is also in positive correlation with the average value of the splay and bend flexoelectric coefficients [1]. If the voltage is beyond a critical threshold such that the dielectric effect is electrically induced, the helix of the CLC with positive dielectric anisotropy is gradually unwound with ascending voltage amplitude, and the LC configuration is eventually sustained in the helix-free homeotropic state at high voltage, leading to the change in effective birefringence from (*n*_e_ + *n*_o_)/2 in the field-off ULH state to *n*_o_ in the voltage-sustained homeotropic state, where *n*_e_ and *n*_o_ are the extraordinary and ordinary refractive indices of the LC, respectively [2]. Owing to the uniqueness, a CLC in the ULH state can be regarded as an electrically tunable birefringent medium with the optic axis along the helical axis in the field-off state for modulating the intensity and phase retardation of polarized light under a pair of crossed polarizers. Since the chiral-flexoelectric effect with sub-millisecond response time was first exploited by Patel and Meyer in 1987 [3], short-pitch CLCs with ULH alignment have drawn a great deal of interest as promising fast-response optoelectronic materials for high-definition displays with a field-sequential color driving scheme, virtual/augmented reality head-mounted elements, and high-speed spatial light modulators in glasses-free 3D displays, as well as light detection and ranging (LiDAR) sensors [4,5,6,7]. Potential applications of ULH with positive dielectric anisotropy based on the voltage-induced dielectric effect have also been suggested as electrically tunable phase retarders [8] and lasers [9].

From the point of view of device fabrication, the formation of a stable and defect-free ULH configuration in a sandwich-type cell is problematic, in that a CLC enclosed in such a simple confining geometry favors stabilization in the Grandjean planar state or the focal conic state to minimize the free energy. The most widely used aligning technique is to apply AC voltage across a cell upon cooling through the isotropic-to-CLC phase transition [3]. The uniformity of ULH formed with this electric field approach has been improved by mechanically shearing the cell substrates simultaneously [10,11] or by modifying the surface alignment conditions with alternate stacking of planar and homeotropic alignment [12], or with helical-pitch-matched cholesteric alignment layer [13]. Other electric field approaches—including those based on voltage-induced electrohydrodynamic instability [14,15], flexoelectric effect [16], textural transition [17], electro-thermal effect [18], and adoption of tri-electrode configurations [19,20]—have been successively proposed for generating ULH alignment in a planar-aligned cell. Nevertheless, most ULH textures as prepared by the above-mentioned voltage pretreatments would be irreversibly destroyed or even transferred back to the most stable Grandjean planar texture after the CLC helix is partially or completely unwound by external voltages or the phase transition often occurs unwantedly by thermal variation; hence, making them incompatible for utilization of the flexoelectric or dielectric switching in extensive electro-optic applications. As such, attention has been paid to adding photocurable precursors into CLCs for forming polymer networks via photopolymerization to effectively stabilize the ULH structure for the flexoelectro-optic effect [2,21,22,23,24,25].

In a practical situation, the flexoelectric coefficients of conventional rod-like LCs are relatively small, so the extent of flexoelectric switching would be insufficient, and the electro-optic characteristics are dominated primarily by the dielectric effect. To promote flexoelectro-optic performance, specific bent-core and bimesogenic LCs with inherently high flexoelectric coefficients and low dielectric anisotropy have been synthesized to lower the operating voltage and enlarge the voltage-driven deviation angle of the optic axis [11,21,26,27]. While these bimesogenic compounds are not as ubiquitous or universal as rod-like LCs and their LC mesophases exist mostly at high temperatures, pronounced flexoelectric effect has alternatively been revealed at room temperature in a binary CLC mixture consisting of bent-core bimesogens incorporated into a commercial rod-like CLC with low dielectric anisotropy [16,22,28]. As the material properties required are a trade-off, it seems difficult to embrace comparably strong flexoelectric and dielectric effects in a CLC ULH cell.

The bent-core LC dimer CB7CB, offering alluring material properties of high flexoelectric coefficients and low bend elastic constant, is the first bimesogneic compound discovered to exhibit the twist-bend nematic phase [29]. More attractively is the fact that mixing CB7CB with a given rod-like CLC has been proven unique, allowing the resultant binary CLC mixture to exhibit the desired flexoelastic ratio in a wide temperature range. Superior features based on CB7CB-doped CLCs have been suggested for a diverse range of photonic and electro-optic applications, especially fast and pronounced flexoelectro-optic responses in the ULH state of CB7CB-doped short-pitch CLCs with promoted flexoelectric coefficients [21,22] and the electrically tunable reflection bandgap in a wide range (from UV to visible and infrared) in the heliconical state of a CB7CB-doped long-pitch CLC whose elastic ratio of bend to elastic constants smaller than 1 [30]. Recently, we have developed a binary CLC mixture containing a bimesogenic LC dimer (CB7CB) with large flexoelectric coefficients, a conventional rod-like nematic LC (E7) with large dielectric anisotropy, and a chiral dopant. By means of dielectric spectroscopy and transmission spectroscopy, as well as textural observations, we have established an electrical approach to generating a stable ULH texture and clarified the unusual frequency-modulated textural switching mechanism in terms of the frequency-*dependent* flexoelectric effect and frequency-*independent* dielectric effect. In the present work, we refer to the alluring material feature specific to the CB7CB-doped E7 CLC and aimed to develop a polymer-network (PN)-stabilized ULH texture for implementing frequency-modulated electro-optic responses via the dielectric and flexoelectric effects. Unlike previously developed polymer networks employing a photocurable monomer with particular ultraviolet (UV) exposure conditions or with high concentrations [2,21,22,23,24,25], the proposed one in this study was constructed by a monomer mixture containing a bifunctional monomer RM257 and a trifunctional monomer TMPTA, permitting the ULH texture to be highly stable and reversibly recoverable at a low concentration of 5-wt% in a total. So far, the polymer system involving the copolymerization of RM257 and TMPTA has widely been applied to the stabilization of blue phases in wide temperature ranges [31,32], but it has not been adopted for stabilizing the ULH texture until now. To demonstrate the unique combination of RM257 and TMPTA, another PN-ULH texture with RM257 alone was fabricated as a comparative counterpart. The stability in the static state and recoverability after stimuli-driven textural or phase transition of the two PN-ULH textures with distinct polymer structures were discussed by textural observation along with transmission and dielectric measurements. Furthermore, the frequency dependence of the flexoelectric and dielectric strengths was manifested by the real-part dielectric spectra. Voltage and frequency-dependent transmission spectra were revealed to clarify the electro-optic responses of the PN-ULH in specific frequency ranges and to demonstrate the feasibility for complying with light intensity and phase modulations by the voltage amplitude and frequency.

## 2. Materials and Methods

### 2.1. Materials

**Figure 1:**
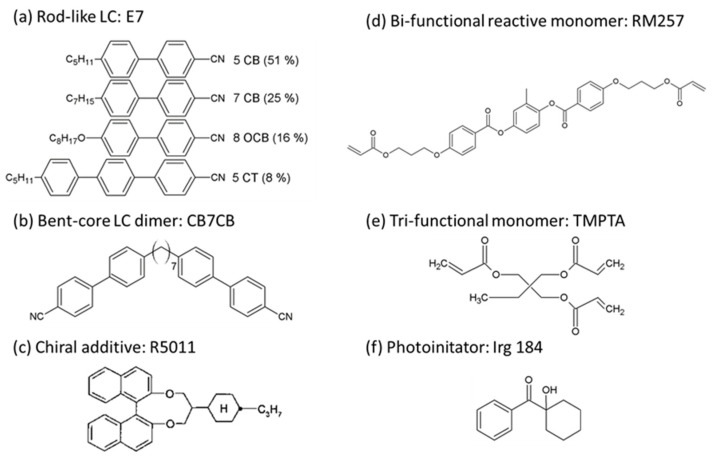
Chemical structures of (**a**) E7, (**b**) CB7CB, (**c**) R5011, (**d**) RM257, (**e**) TMPTA, and (**f**) Irg 184.

Materials used in this study include the calamitic nematic LC E7, bent-core LC dimer CB7CB (1″,7″-bis(4-cyanobiphenyl-4′-yl)heptane), chiral additive R5011, photocurable monomers RM257 (1,4-Bis-[4-(3-acryloyloxypropyloxy)benzoyloxy]-2-methyl-benzene) and TMPTA (1,1,1-trimethylolpropane triacrylate), and the photoinitiator Irg184 (1-hydroxy cyclohexane benzophenone). E7 was provided by Daily Polymer Corp., Kaohsiung, Taiwan, and TMPTA was purchased from Alfa Aesar, Ward Hill, MA, USA. The others were obtained from Jiangsu Hecheng Display Technology Corp., Nanjing, China. All the above-mentioned materials with chemical structures, as illustrated in Figure 1, were used as received without further purification. E7 with a rod-like molecular shape is a cyano-based and four-component eutectic mixture with a clearing temperature *T_c_* = 59 °C, birefringence Δ*n* = 0.225 (measured at the wavelength *λ* = 589 nm and temperature *T* = 20 °C), and positive dielectric anisotropy of Δ*ε* = +14.3 (measured at the frequency *f* = 1 kHz and *T* = 20 °C), as given in the datasheet. CB7CB is a bent mesogenic dimer whose material properties have been found particularly to exist as a nematic twist-bend phase (N_TB_) at *T* < 99 °C [33], showing a high flexoelectric coefficient of ~31 pC/m and low dielectric anisotropy around +1–2 [21]. R5011 exhibits right-handed chirality with a relatively high helical twisting power (HTP) over 100 μm^−1^. RM257 is a bifunctional liquid crystalline monomer with a rod-like molecular shape, and TMPTA is a trifunctional monomer with three acrylate ester groups. The function of the photoinitiator Irg184, capable of generating free radicals under UV exposure, is to accelerate the photopolymerization process of monomers.

### 2.2. Sample Preparations

A binary achiral LC mixture, consisting of 55-wt% E7 and 45-wt% CB7CB, was blended with R5011 at a weight percentage of *c*_R5011_ = 3.8-wt% to obtain a short-pitch CLC with a reflective bandgap lying in the UV light regime in the Grandjean planar state and with bimesogen-enhanced flexoelectro-optic responses in the ULH state. The HTP of R5011 in the 45-wt% CB7CB-containing E7 was proven to be 145 μm^−1^ [16] so that the helical pitch length of the resultant CLC is 181 nm as calculated by (HTP × *c*_R5011_)^−1^. Two monomer/CLC precursors, designated RM50 and RM25TM25, were prepared by incorporating the CLC with 5.0-wt% RM257 and 0.2-wt% Irg 184, and with 2.5-wt% RM257, 2.5-wt% TMPTA, and 0.2-wt% Irg 184, respectively. After thorough stirring for 2 h at 100 °C to allow homogeneous blending. Each monomer/CLC precursor in the isotropic phase at 100 °C was then injected via the capillary action into a planar-aligned cell (Chiptek, Miaoli, Taiwan) with a cell gap of 5.0 ± 0.5 μm and overlapped electrode area of 0.25 cm^2^. The inner surfaces of the two substrates were spin-coated with the polyimide DL-2360 as the planar aligning agent and rubbed in anti-parallel directions to impose the planar surface anchoring of LC molecules with a pretilt angle of 4–6°. The phase sequences of RM25TM25 and RM50 precursors as examined by optical textures in the cooling process from 70 °C to 0 °C are Isotropic—58 °C—blue phase—17 °C—CLC—7 °C—SmA* and Isotropic—61 °C—blue phase—18 °C—CLC—11 °C—SmA*, respectively. Note that the obtained blue phase within a temperature range over 40 °C is not permanently stable because it would transfer to the CLC phase after applying a significantly high voltage to unwind the helical structure or by heating from a temperature in the SmA* phase.

### 2.3. Formation of a Polymer-Network Uniform Lying Helix (PN-ULH) Texture

**Figure 2:**
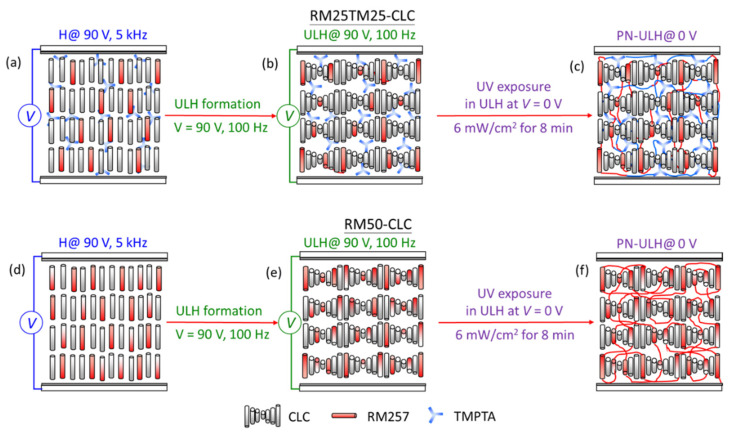
Schematic illustrations of steps for fabricating a PN-ULH texture in (**a**–**c**) an RM25TM25-CLC cell and (**d**–**f**) an RM50-CLC cell. (**a**,**d**) Electrically sustaining LC orientation by a 90-V_rms_ voltage at 5 kHz; (**b**,**e**) forming ULH texture by switching the frequency of 90-V_rms_ voltage from 5 kHz to 100 Hz; (**c**,**f**) constructing polymer network via photopolymerization after 8 min of 6 mW·cm^−2^ UV exposure.

Figure 2 illustrates the procedure for forming a given polymer structure in an RM25TM25-CLC or an RM50-CLC cell with ULH alignment. All steps were performed at T = 25 °C stabilized with a temperature controller (Linkam Scientific, T95-PE, Surrey Tadworth, UK). First, a 90-V_rms_ voltage at 5 kHz was applied across the cell to sustain the LC orientation in the homeotropic state (Figure 2a,d). Next, referring to the established electric field approach in our previous work [16], defect-free, monodomain ULH alignment was electrically induced via the flexoelectric effect by switching the frequency of 90-V_rms_ voltage directly from 5 kHz to 100 Hz (Figure 2b,e). By decreasing the voltage slowly from 90 V_rms_ to 0 V_rms_ to avoid defect generation, the well-aligned ULH texture was stably preserved, and the cell at zero voltage was illuminated with UV light at λ = 365 nm and intensity of 6 mW·cm^−2^ derived from a Panasonic Aicure UJ35 LED spot-type UV curing system to allow chain polymerization of monomers. After 8 min of UV exposure, two PN-ULH textures were obtained with distinct polymer structures constructed by 2.5-wt% RM257 and 2.5-wt% TMPTA in the RM25TM25-CLC cell (Figure 2c) and by 5.0-wt% RM257 in the RM50-CLC cell (Figure 2f).

### 2.4. Measurements

Measurements were performed at *T* = 25 °C unless specified. The types of mesophases and CLC textures were identified by optical images using a polarizing optical microscope (BX51-P, Olympus, Tokyo, Japan) in transmission mode with a 10× objective lens. Images were taken by a digital camera (Olympus XC30, Tokyo, Japan) with a resolution of 2080 × 1544 pixels mounted on the microscope. To evaluate optical and electro-optic characteristics of a cell, transmission spectra in the visible range of 400 nm–750 nm were acquired with a high-speed fiber-optic spectrometer (Ocean Optics HR2000+, Shanghai, China) in conjunction with a halogen light source (Ocean Optics HL2000, Shanghai, China). Time-dependent transmission features were monitored using a He–Ne laser operating at *λ* = 632.8 nm as the light source. All the experiments mentioned above were accomplished under crossed polarizers, and the angle between the helical axis of ULH in the field-off state and the transmission axis of either polarizer is defined as the polarizing angle *α*. An arbitrary function generator (Tektronix AFG-3022B, Beaverton, OR, USA) was connected to an amplifier (TREK Model 603, New Taipei, Taiwan) to permit electric field (in square waves) applied across a cell in a wide tunable range of 0 V–125 V. Dielectric spectra were obtained using a precision LCR meter (Agilent-E4980A, Santa Clara, CA, USA) with a measurable frequency range from 20 Hz–2 MHz. The probe voltage was as low as 0.5 V_rms_ to avoid reorienting LC molecules in the dielectric measurement.

## 3. Results and Discussion

### 3.1. Stability and Recoverability of PN-ULH Textures

**Figure 3:**
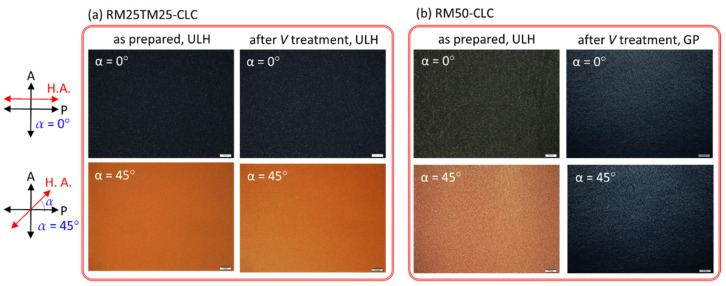
Optical images under crossed polarizers in the static state at *α* = 0° and 45° of (**a**) RM25TM25-CLC (**b**) RM50-CLC as prepared without any prior stimulus and after a voltage treatment at *T* = 25 °C. The voltage treatment entails a 100-V_rms_ voltage at 5 kHz across a cell. Scale bar: 50 μm. P, A and H.A. represent the orientations of the transmission axis of the linear polarizer, the analyzer, and the ULH helical axis, respectively.

**Figure 4:**
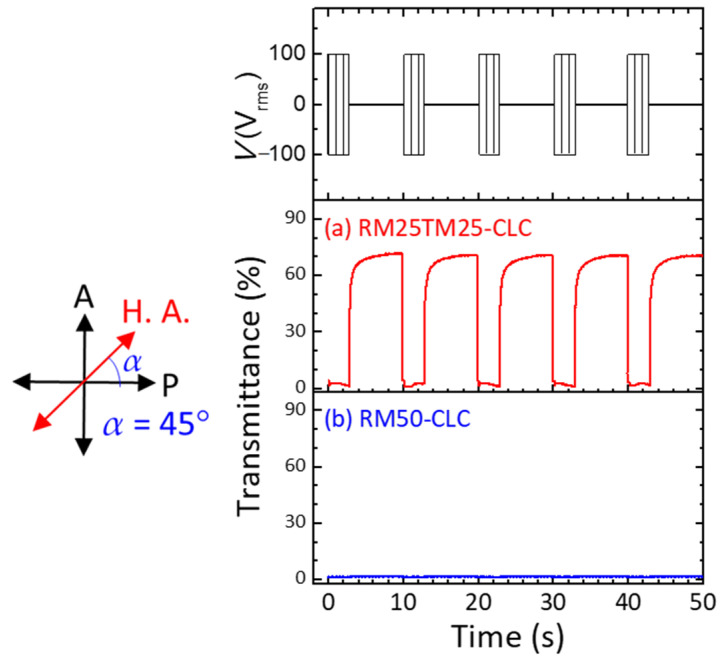
Dynamic transmission at *λ* = 632.8 nm of (**a**) RM25TM25 and (**b**) RM50 CLC cells (azimuthally oriented at 45° between crossed polarizers) subjected to voltage pulses consisting of *V* = 100 V_rms_ at 5 kHz for 3 s and *V* = 0 for 7 s successively in a pulse.

Figure 3 shows optical textures in the static state of the RM25TM25- and RM50-CLC cells as prepared and after treating with a cycle of voltage-induced helical unwinding. In the case of the RM25TM25-CLC (Figure 3a), the uniform dark image at *α* = 0° and bright image at *α* = 45°, attributable to the birefringent effect, indicate that the PN-ULH texture thus-formed following the said procedure in Figure 2a–c was stable and of high quality with a unidirectional helical axis parallel to the rubbing direction. By applying a significantly high voltage at *V* = 100 V_rms_ and *f* = 5 kHz across the cell thickness, enabling the ULH helix to be unwound and LC orientation sustained in the homeotropic state, the dark (bright) image at *α* = 0° (45°) at *V* = 0 V was virtually unchanged after the voltage removal (Figure 3a), connoting that the PN-ULH was retained as it was prior to the voltage application. In comparison, the RM50-CLC as prepared revealed a stable PN-ULH texture as implied by the appearance of high dark-to-bright contrast between images at *α* = 0° and 45° in Figure 3b. For cells prepared because more stripe defects were generated during the generation of ULH in RM50-CLC step prior to photopolymerization, the optical texture of the resulting PN-ULH (Figure 3b) is somewhat non-uniform as compared with that of RM25TM25-CLC (Figure 3a). Nevertheless, when a cycle of helical unwinding and rewinding process was performed by the switching of a 5-kHz voltage from 100 V_rms_ to 0 V, the Grandjean planar texture instead of ULH was generated together with those already existing stripe defects at V = 0 V, leading to equally dark optical images with visible tiny-sized domains at α = 0° and 45° (Figure 3b). The validity of the Grandjean planar texture in the RM50-CLC sample after voltage treatment has been double-checked by the visible light transmission spectrum without any polarizer, which shows transmittance of ~80% on average compared to that of ITO glass used (data not shown). The resultant Grandjean planar texture with high transparency in visible light might be attributable to the CB7CB-doped CLC host with matched bend and twist elastic constants so that the nucleation process for the transition from the transient planar state to the initial Grandjean state was reduced [34]. Subsequently, a continuous voltage waveform comprising *V* = 100 V_rms_ at 5 kHz for 3 s and V = 0 for 7 s in a period was designated to repeatedly unwind and rewind the CLC helix. Figure 4 depicts the time-varying optical transmission (*λ* = 632.8 nm) of the CLC cells in response to the voltage wave train. The reliability and recoverability of the PN-ULH in the RM25TM25-CLC cell can be ascertained by the results in Figure 4a, showing equally high contrast between transmittances at ~1% in durations with *V* = 100 V_rms_ at 5 kHz (i.e., the *V*-sustained H state) and ~70% in those with *V* = 0 V (i.e., the stable ULH state) in every period. It is worth noting that the PN-ULH in the RM25TM25-CLC after cycles of voltage treatments can stably be maintained for at least 2 weeks. In contrast, Figure 4b indicates that driving the RM50-CLC with the designated voltage waveform resulted in nearly invariant transmittance at ~1% over time. Because the optic axes in the Grandjean planar and homeotropic states were perpendicular to the substrate plane and along the propagation direction of normal incidence of light, no phase retardation was accumulated, and the intensity of light passing through a CLC cell in either state under crossed polarizers principally vanished. Consequently, it is suggested that the helices in the RM50-CLC, firstly unwound by the 100-V_rms_ voltage, tended to rewind to form the Grandjean planar texture rather than the ULH after removing the voltage. Although the ULH configuration in the RM50-CLC can be electrically regenerated following the steps illustrated in Figure 2d,e, it was still unstable after a cycle of helical relaxation from unwinding to rewinding. The results described above support that the polymer framework constructed by the copolymerization of 2.5-wt% RM257 and 2.5-wt% TMPTA was significant, allowing the ULH texture to be optically stable in the static state, electrically switchable to the homeotropic state, and recoverable from the helical unwinding state. However, for the counterpart with the TMPTA content fully replaced by RM257, the polymer structure made from the bifunctional reactive monomer alone (at 5.0-wt%) in the RM50-CLC became insufficient to sustain the ULH helix, causing it to become unstable after the CLC helix was unwound. This underlines the importance of TMPTA, which has been found to play the role of promoting the crosslinking density and introducing rigidity to the resulting polymer network with RM257 [31,32].

**Figure 5:**
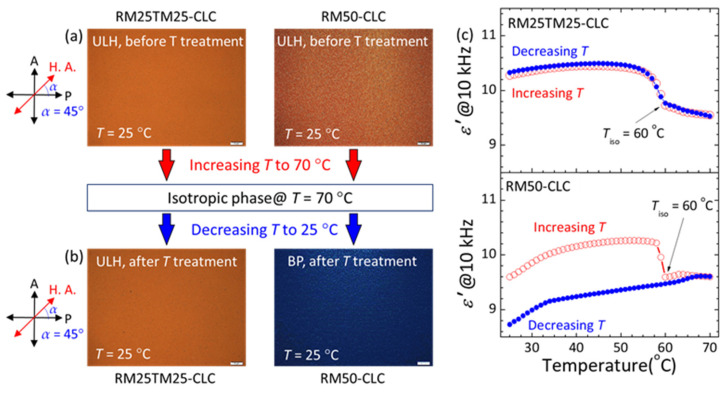
Optical images under crossed polarizers at *α* = 45° and *T* = 25 °C of RM25TM25-CLC RM50-CLC cells (**a**) before and (**b**) after a cycle of thermal treatment. (**c**) Temperature-dependent real-part dielectric permittivity at *f* = 10 kHz of the two cells, as measured by first increasing *T* from 25 °C to 70 °C, followed by decreasing *T* back to 25 °C with a heating/cooling rate of 1 °C/min. The subscript “iso” represents “isotropic.” Scale bar: 50 μm.

Furthermore, the thermal stability of PN-ULH textures and their recoverability after thermally induced phase transition were assessed in accordance with simultaneous measurements of optical images and real-part dielectric permittivity (*ε*′) at varying temperatures as shown in Figure 5. Here, the ULH texture in the RM50-CLC was regenerated following the electric field approach illustrated in Figure 2d,e. The two cells initially with PN-ULH textures were first heated progressively from 25 °C to 70 °C at a rate of 1 °C/min. As they were unraveled by the bright appearances in the optical images at *α* = 45° (e.g., Figure 5a at 25 °C), the PN-ULH textures in this heating process were preserved until *T* > *T*_iso_ = 60 °C, where the CLCs were in the isotropic phase with dark appearances in optical images (data not shown). The value of *ε*′ in the partially ordered CLC ULH state, equaling to (*ε*_||_ + *ε*_⊥_)/2, is greater than (*ε*_||_ + 2*ε*_⊥_)/3 in the disordered isotropic phase, where *ε*_||_ and *ε*_⊥_ are respectively the parallel and perpendicular components of dielectric permittivity of LC molecules.

The CLC-to-isotropic phase transition behavior upon heating can be depicted by the decreasing *ε*′ with elevating *T*, particularly in the temperature range of 56–60 °C (red open circles in Figure 5c), and the exact value of *T*_iso_ = 60 °C be quantified by the first-order derivative of *T*-dependent *ε*′. When the cells were subsequently cooled down from 70 °C to 25 °C, the PN-ULH configuration in the RM25TM25-CLC cell was recovered with an identical bright texture, but in RM50-CLC it disappeared and, instead, the blue phase with a dark-blue texture emerged after the transition from the isotropic phase at *T* < *T*_iso_ = 60 °C (Figure 5b). Therefore, the *T*-dependent *ε*′ curves, measured in the heating and cooling processes, overlapped in the RM25TM25-CLC but behaved differently in the RM50-CLC (Figure 5c). It is worth mentioning that the PN-ULH in the RM25TM25 cell could also be retained after heating from the SmA* to CLC phase. While for the blue phase in the RM50 cell, it was still unstable and became a Grandjean planar CLC by electrically unwinding the helical structure or by thermally induced phase transition from the SmA* phase.

**Figure 6:**
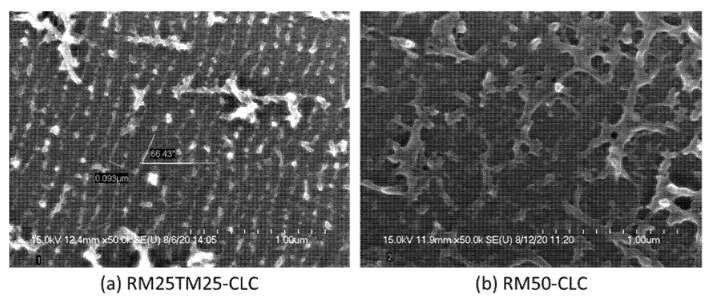
SEM images from a top-view of polymer network morphologies on a substrate surface of (**a**) RM25TM25 and (**b**) RM50 CLCs with PS-ULH textures.

To inspect morphologies of the polymer structures in the two CLCs, each cell was opened, and the two substrates were rinsed with acetone to remove LCs and then subjected to a scanning electron microscope (SEM; Hitachi S-4700 Type II, Tokyo, Japan). Figure 6 shows SEM images from the top-view of one of the two substrates of each PN-ULH CLC cell. One can clearly identify that a periodical polymer network was obtained on the surface of the RM25TM25 cell with a regular periodicity of ~96 nm equal to half of the helical pitch of the CLC host used (Figure 6a), but no such a periodical morphology was found at the substrate surface of the RM50-CLC (Figure 6b). The same result regarding the existence of a surface-localized polymer network was observed for the other substrate of a cell. With fixed UV exposure conditions (i.e., 6 mW·cm^−2^ for 8 min) in this work, the polymer structure stemming from 5.0-wt% RM257 was primarily formed in the main, but from 2.5-wt% RM257 and 2.5-wt% TMPTA was distributed not only in volume but at the surface. So far, depending on the constituents of the CLC hosts and the UV exposure conditions, a good number of studies have demonstrated stable PN-ULH textures enabled by polymer networks locally at one or two substrate surfaces [21,22], uniformly in volume, non-uniformly in volume, and at surfaces [2,23,24], or even with polymer walls [25] by using a kind of reactive monomer, such as RM257 or BAB6, in a wide span of concentrations between 3.5-wt% and 25-wt%. It is likely that for the binary CB7CB/E7 CLC host used in this study, a stable and recoverable PN-ULH texture could be created by increasing the concentration of RM257 over 5.0-wt%, but the trade-off would be the degraded electro-optic performance, especially at the expense of the increased operation voltage and residual birefringence. As a result, the RM25TM25-CLC with a stable and recoverable PN-ULH texture in a wide temperature range was adopted to explore electro-optic responses based on the flexoelectric and dielectric effects in the next section.

### 3.2. Frequency-Modulated Electro-Optic Responses

**Figure 7:**
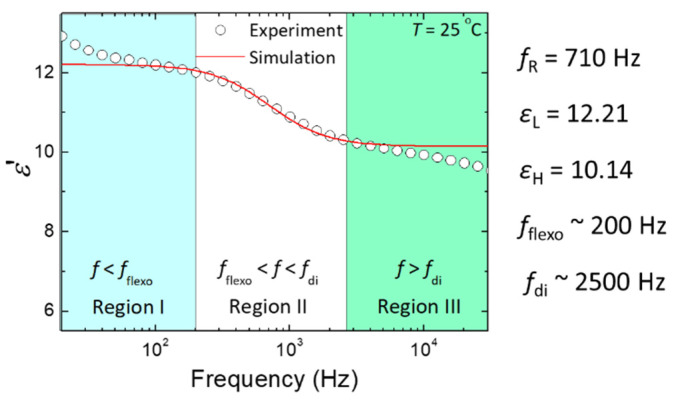
Real-part dielectric spectra *ε*′(*f*) of the RM25TM25-CLC cell in the PN-ULH state at *T* = 25 °C. The simulated *ε*′(*f*) curve (solid red line) is plotted with fitting parameters of *f*_R_ = 710 Hz, *ε*_L_ = 12.21 and *ε*_H_ = 10.14, as deduced by fitting the experimental data (open circles) into Equation (1).

Prior to electro-optic investigations, we first discussed the extent of the flexoelectric effect, modified by the bimesogen CB7CB in the RM25TM25 CLC, via the real-part dielectric spectrum (*ε*′(*f*)) of the cell in the PN-ULH state according to the flexoelectric contribution to the dielectric permittivity. For a CLC in the ULH state with a strong flexoelectric effect, two types of dielectric relaxation behavior by the coupling of molecules to the electric field can be induced in distinct frequency regimes. The primary is connected to the orientational polarization by the molecular rotation around the short axis, which typically occurs beyond MHz in conventional thermotropic LCs and thus is excluded in this study. The secondary from chiral-flexoelectric polarization with a relaxation frequency between several hundred hertz and a few kilohertz is attributable to the splay-bend distortion through flexoelectric coupling to the electric field [28]. As plotted in Figure 7, the resolved frequency dispersion of dielectric permittivity with a relaxation frequency at ~710 Hz in the frequency range of 20 Hz–30 kHz from the experimental data (open circles) is undoubtedly attributable to the flexoelectric polarization, which has been theoretically explained in [28] and experimentally demonstrated in [16]. Such a Debye-like dielectric relaxation behavior contributed by flexoelectric polarization can be expressed in terms of the relaxation frequency (*f*_R_), dielectric permittivities at low- (*ε*_L_) and high-frequency (*ε*_H_) limits as:

(1) $$\varepsilon \prime (f)=\varepsilon_{H}+\frac{\varepsilon_{L}-\varepsilon_{H}}{1+{\left(f/f_{R}\right)}^{2}}$$

By fitting the experimental data of Figure 7 to Equation (1), we obtained *f*_R_ = 710 Hz, *ε*_L_ = 12.21 and *ε*_H_ = 10.14. The dielectric strength (*ε*_flexo_ ) of this relaxation proportional to *e*^2^/*K* [28] is equal to 2.07 as calculated by *ε*_flexo_ = *ε*_L_ − *ε*_H_, where *e* and *K* are average values of splay and bend flexoelectric coefficients and elastic constants, respectively. Using these fitting parameters and referring to Equation (1), the original profile of the dielectric relaxation was simulated and plotted in Figure 7 (solid red line), which agrees with the experimental data. Note that the slight increase (decrease) of dielectric permittivity with decreasing (increasing) frequency at *f* < 100 Hz (*f* > 4 kHz) in the experimental dielectric spectrum is attributable to the induction of another dielectric relaxation from space-charge polarization via ion transport (non-ideal cell geometry with finite conductivity of ITO electrodes). Because the flexoelectric switching is polar, the magnitude of the flexoelectric response of LC molecules would be a function of the frequency of the AC electric field. This allows us to divide the dielectric spectrum in Figure 7 into three frequency regimes by two designated frequencies of *f*_flexo_ = 200 Hz at *ε* ≡ *ε*_L_ − 0.1*ε*_flexo_ and *f*_di_ = 2500 Hz at *ε* ≡ *ε*_H_ + 0.1*ε*_flexo_ to characterize the frequency dependence of flexoelectric switching. As specified in [16], the flexoelectric effect is significant and independent of the frequency in region I (*f* < *f*_flexo_ = 200 Hz), but it is completely suppressed in region III (*f* > *f*_di_ = 2500 Hz).

**Figure 8:**
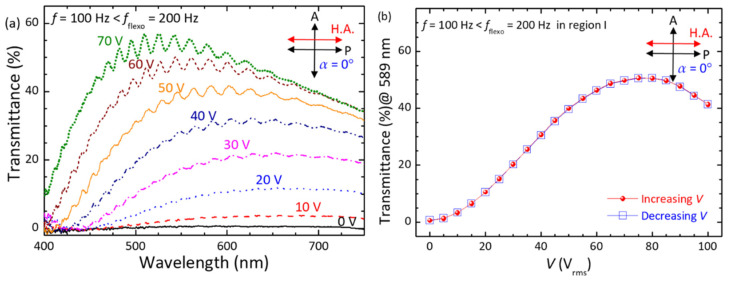
(**a**) Transmission spectra of the RM25TM25-CLC cell in the PN-ULH state driven by various voltages at a fixed frequency of 100 Hz. (**b**) Voltage-dependent transmission curves at *λ* = 589 nm and *f* = 100 Hz in the increasing- and decreasing-voltage processes. The cell for measurements at *T* = 25 °C was placed between crossed polarizers at a fixed polarizing angle of *α* = 0°.

**Figure 9:**
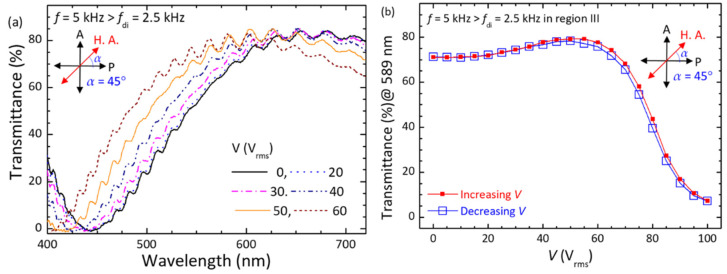
(**a**) transmission spectra of the RM25TM25-CLC cell in the PN-ULH state driven by various voltages at 5 kHz. (**b**) Voltage-dependent transmission curves at *λ* = 589 nm and *f* = 5 kHz upon increasing and decreasing voltage. The cell for measurements at *T* = 25 °C was placed between crossed polarizers at a fixed polarizing angle of *α* = 45°.

Based on the frequency regions defined, optical properties of the cell in the PN-ULH state driven by voltages at *f* = 100 Hz in region I (Figure 8) or 5 kHz in region III (Figure 9) were discussed by measuring transmission spectra in the wavelength range of 400 nm–750 nm. According to Figure 7, the onset frequency for space-charge-polarization-induced dielectric relaxation is lower than 100 Hz. Our pre-tests ensured that no helical CLC textures could be electrically induced in the investigated frequency regime (100 Hz–5 kHz), such as Grandjean planar and focal-conic in general by the dielectric effect, and dynamic scattering, stripe, and Williams domains in particular via the ionic effect. Here, the cell was situated between a pair of crossed polarizers; thus, the voltage-dependent intensity of light propagating through the birefringent ULH texture can be written as:

(2) $$I=I_{0}\sin^{2}\left\{2\left[\alpha +\phi (V)\right]\right\}\sin^{2}\left\{\frac{\pi d}{\lambda }\left[n_{\mathrm{eff}}\left(V\right)-n_{o}\right]\right\}$$

where *I*_0_ is the intensity of incident light, *α*, as defined earlier in the Experimental section, is the angle between the transmission axis of a polarizer and the ULH helical axis at *V* = 0 V, *λ* is the wavelength of the incident light, *ϕ* (*V*) is the voltage-induced in-plane deviation angle of the ULH helical axis by the flexoelectric effect, and *n*_eff_ (V) as the effective refractive index is a function of the voltage-induced helical deformation by the dielectric effect. In the field-off state (*V* = 0 V), *ϕ* (*V* = 0 V) = 0° and *n*_eff_ (*V* = 0 V) = (*n*_e_ + *n*_o_)/2 so that the transmittance is zero at α = 0° (Figure 8a) and the optical spectrum at *α* = 45° exhibits wavelength dispersion of transmission with a peak transmittance of *T*%_peak_ ~84% at *λ*_peark_ ~650 nm and a minimal transmittance of *T*%_min_ ~0% at *λ*_min_ ~440 nm (Figure 9a). Accordingly, as supported by Equation (2), the magnitude of *T*%_peak_ would be primarily determined by *ϕ* (*V*) as long as the phase retardation in the second term is greater than *π*/2, and *λ*_peark_, as well as *λ*_min_, would be a function of *n*_eff_ (*V*). By setting *α* = 0° and applying a voltage at *f* = 100 Hz (<*f*_flexo_ = 200 Hz in region I) across the cell thickness, the optical transmission profiles as delineated in Figure 8a were strongly dependent on the voltage amplitude, showing increased *T*%_peak_ from ~3.7% at *V* = 10 V_rms_ to ~56% at *V* = 70 V_rms_ and, in the meantime, blue-shifted *λ*_peark_ and *λ*_min_ at *V* > 20 V_rms_. This manifests a varying degree of splay-bend deformation by the flexoelectric effect and helical unwinding by the dielectric effect, induced by voltage at a frequency in region I, giving rise to the modulation of light intensity and phase retardation by changing *ϕ* (*V*) and *n*_eff_ (*V*) simultaneously. Notably, while plotting the voltage-dependent transmission (*V*−*T*%) curves with increasing and decreasing voltages from the optical spectral data, hysteresis-free electro-optic responses with a contrast ratio of ~80 (as calculated by the ratio between maximal transmittance of ~50.7% at 75 V_rms_ and minimal transmittance of ~0.6% at 0 V) were demonstrated (Figure 8b) thanks to the stabilization of the PN-ULH texture with high recoverability in the RM25TM25-CLC cell. The time in response to the voltage at *f* = 100 Hz at *T* = 25 °C, attributed to the combination of flexoelectric and dielectric effect, was on the order of sub-millisecond, varying from 1 ms at *V* = 10 V_rms_ to 0.28 ms at *V* = 70 V_rms_ (data not shown). On the other hand, when the voltage frequency was changed from 100 Hz to 5 kHz (>*f*_di_ = 2500 Hz in region III), it is clear from Figure 9a that the transmission spectra at *α* = 45° with nearly invariant *T*%_peak_ ~84% blue-shifted with ascending voltage amplitude beyond a threshold at ~20 V_rms_. This suggests that the flexoelectric effect is considerably suppressed in the frequency region III, and the optical responses to the external voltage are primarily dominated by the dielectric effect and thereby the change of *n*_eff_ (*V*) with the voltage strength. The *V*−*T*% curves in Figure 9b denote that the electro-optic responses characterized by the dielectric effect at *f* = 5 kHz in region III were hysteresis-free as well, supporting again that the PN-ULH can be completely recovered after the CLC helix is partially or fully unwound by the applied voltage. Deduced from the results in Figure 9b, the threshold voltage for unwinding the helix was ~20 V_rms,_ and the voltage for sustaining LC molecules in the homeotropic state was ~100 V_rms_.

**Figure 10:**
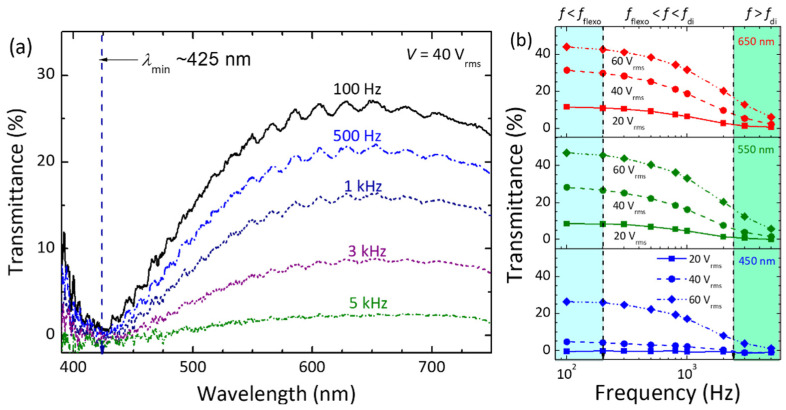
(**a**) Transmission spectra of the RM25TM25-CLC cell in the PN-ULH state driven by *V* = 40 V_rms_ at various frequencies. (**b**) Frequency-dependent transmission at *λ* = 450 nm, 550 nm, and 650 nm of the cell applied with *V* = 20 V_rms_, 40 V_rms_, and 60 V_rms_, respectively. Measurements were performed with crossed polarizers at *α* = 0°.

By fixing the voltage amplitude and permitting the frequency as a variable, an unusual frequency-modulated electro-optic feature, demonstrating electrically tunable light intensity without phase shift, was initiated for the first time to the best of our knowledge. This is evidenced by the results shown in Figure 10a using *V* = 40 V_rms_ as the example, which yielded invariant *λ*_min_ at ~425 nm and a gradual decrement in *T*%_peak_ with increasing frequency. It has been ensured that the intense frequency-dependent optical characteristics can also be realized at an arbitrary voltage in the range of 20 < V < 100 V_rms_ with a varying degree of tunable light intensity range (Figure 10b) but the value of *λ*_min_ as a constant blue-shifted with increasing voltage. The underlying mechanism can be explained by the frequency dependence of the flexoelectric effect and frequency-independent dielectric effect in the frequency regime featuring the flexoelectric-polarization-dominated dielectric relaxation. The dispersion of transmittance of *f*–*T*% curves in Figure 10b is analogous to that of the dielectric permittivity of the dielectric spectrum in Figure 7. Consequently, frequency-dependent electro-optic responses reported in this work are beneficial. This advantage enables the dual operation of the proposed bimesogen-doped CLC cell with a stable PN-ULH texture in the amplitude mode for controlling the light intensity without phase shift by the voltage *frequency* (based on the voltage-induced flexoelectric and dielectric effects) and in the phase mode for modulating the phase retardation by the voltage *amplitude* at a fixed frequency in region III (according to the dielectric effect). It is worth noting here that the dielectric relaxation and frequency-modulated electro-optic responses obtained in the PN-ULH are attributable to the CLC host with a significant flexoelectric effect due to the incorporation of CB7CB. Therefore, all the results demonstrated in this section would be enabled or reproducible by using other bent-core bimesogens if they can play the role of the main component in the explored CLC mixture to obtain the bimesogen-enhanced flexoelectric effect [28,35].

## 4. Conclusions

We have produced a PN-ULH texture and explored its electro-optic characteristics in a bimesogen-doped CLC. With a fixed monomer concentration of 5-wt% incorporated into the CLC, our experimental results showed that the polymer network structure with 2.5-wt% RM257 and 2.5-wt% TMPTA in the RM25TM25-CLC cell is more sufficient than that with 5.0-wt% RM257 in the RM50-CLC for sustaining the desired ULH texture with high stability in the static state and excellent recoverability after cycles of electrically induced textural transition to the homeotropic state (Figure 3 and Figure 4) and thermally induced phase transition to the isotropic phase (Figure 5). Evidence from the SEM images inferred that the copolymerization of RM257 and TMPTA promotes the generation of periodic polymer networks at both substrate surfaces with a periodicity of ~96 nm, which equals the half-pitch of the CLC (Figure 6).

Focusing on the PN-ULH texture in the RM25TM25-CLC, frequency-dependent flexoelectric effect, originating from the bimesogen-doped CLC host, was characterized by the real-part dielectric spectrum (Figure 7). In accordance with the profile of dielectric relaxation induced by the flexoelectric polarization, two frequency regions of *f* < *f*_flexo_ = 200 Hz (region I) and *f* > *f*_di_ = 2500 Hz (region III), as specified with the pronounced and suppressed flexoelectric effect, respectively, were defined to investigate the effect of voltage frequency on the electro-optic responses of the PN-ULH texture. Results of the voltage-dependent transmission spectra indicated that the light intensity is electrically tunable together with the blue-shift of the optical spectrum (transmission minimum in Figure 8a) with increasing voltage at *f* = 100 Hz in region I because of the induction of flexoelectric and dielectric effects. By varying the frequency to 5 kHz in region III, LC molecules were unable to respond to the electric field for flexoelectric switching so that only the phase-shift effect was obtained by the dielectric coupling of LCs with the voltage (Figure 9a). It is worth noting based on hysteresis-free *V*−*T* curves in Figure 8b and Figure 9b that, particularly due to the frequency-dependent flexoelectric effect and frequency-independent dielectric effect in the frequency range highlighted by the flexoelectric-polarization-induced dielectric relaxation, a superior feature, enabling frequency modulation of light intensity without phase shift, was implemented (Figure 10). Accordingly, the proposed bimesogen-doped CLC, featuring the highly stable ULH texture by polymer stabilization and the alluring function of frequency-modulated electro-optic characteristics, paves a new pathway toward developing a dual (amplitude and phase modulation)-mode electro-optic device for potential applications in information displays, phase retarders, spatial light modulators, and other optical and photonic devices. For the proposed bimesogen-doped CLC system, miscible bent-core and rod-like LCs are typically polar in nature, and a strong flexoelectric effect is induced generally by low-frequency voltages. Further discussion on the issue of the impact of ionic effect on the electrical and electro-optic characteristics (e.g., the threshold voltage, hysteresis, response time, and voltage holding ratio) is essential for advancing technical applications.

## Data Availability

The authors confirm that the data supporting the findings of this study are available within the article.
